# Reporting and Specifying the Implementation Strategies Used in a National Programme of Primary Care Youth Mental Health

**DOI:** 10.1111/eip.70147

**Published:** 2026-04-07

**Authors:** Jeff Moore, Gillian O'Brien, Sarah Cullinan, Joseph Duffy

**Affiliations:** ^1^ Jigsaw—the National Centre for Youth Mental Health Dublin Ireland; ^2^ University College Dublin, School of Psychology Dublin Ireland

**Keywords:** early intervention, implementation strategies, youth mental health

## Abstract

**Background:**

The implementation strategies used to facilitate the delivery of youth mental health interventions are rarely reported. Specifying and reporting implementation strategies for impactful innovations that have demonstrated good clinical outcomes has the potential to support replication in other contexts and accelerate the adoption of effective youth mental health interventions.

**Methods:**

Key project personnel conducted a retrospective review of implementation strategies used in a national programme of enhanced primary care youth mental health services. These strategies were classified and reported using the pragmatic implementation‐reporting tool. We used univariate statistics to describe the implementation strategy types, temporality, dosage and actors. We conducted linear regression techniques to predict time investment.

**Results:**

We identified 30 implementation strategies used across the course of one year. Twenty percent of strategies identified were in the domains of evaluative and iterative strategies and stakeholder engagement. This was followed by training and educating stakeholders, providing interactive assistance and supporting clinicians. In terms of dosage, interactive assistance accounted for over half of all time invested. Interactive assistance was the only category to predict increased person hours.

**Conclusions:**

By specifying and reporting the implementation strategies used in a youth mental health primary care programme, the pragmatic implementation‐reporting tool can help demonstrate resourcing and skills required to reach programme sustainment.

## 1 Introduction

Adolescence and early adulthood are periods when mental health difficulties commonly emerge (Kessler et al. 2005). Access to evidence‐based interventions (EBIs) can reduce this burden, but many young people still do not receive timely care (Dixon and Patel 2020). Integrated youth mental health programmes such as Headspace in Australia, Jigsaw in Ireland and ACCESS Open Minds in Canada aim to improve access to timely, youth‐centred, evidence‐based support (WEF 2020; Hetrick et al. 2017). These services show strong clinical and economic outcomes (Moore et al. 2025; McGorry et al. 2024; Rickwood et al. 2023), yet the strategies used to implement and sustain them are rarely reported, limiting replication and scalability (Novins et al. 2013; Powell et al. 2014; Kohn et al. 2004).

Implementation science aims to provide tools and knowledge to help move effective programmes into routine use (Proctor et al. 2009; Dixon and Patel 2020; Damschroder et al. 2009) and is central to scaling up effective youth mental health initiatives and reducing the treatment gaps (Zolfaghari et al. 2022; Fixsen et al. 2015). Implementation strategies (ISs) are core to this science, describing techniques used to enhance the uptake and sustainment of innovations (Proctor et al. 2013; Powell et al. 2015). Despite growing attention, ISs are often inconsistently reported, limiting their replicability and systematic testing (Hooley et al. 2020; Proctor et al. 2013). There also remains limited evidence on which strategies best support sustainment, particularly in youth mental health (Shelton et al. 2018; Nathan et al. 2022; Zolfaghari et al. 2022).

This article sets out to report the ISs used in delivering a national youth mental health primary care service in the Republic of Ireland. Jigsaw is one of only a few enhanced primary care youth mental health services that have been scaled to a national level and is now embedded into the mainstream mental health system. In this naturalistic study, we draw on recent implementation science reporting guidelines (Proctor et al. 2013) to (a) describe the ISs employed in delivering this primary care youth mental health service and (b) summarise implementation over the course of a year.

## 2 Implementation Strategies

ISs comprise a range of activities aimed at making it easier to adopt, implement, sustain and expand innovations (Proctor et al. 2013; Powell et al. 2015). Despite significant progress in implementation science over the past decade, insufficient reporting of ISs has been identified as a major issue for the field (Proctor et al. 2013). Just as vague reporting of clinical interventions limits our understanding, unclear descriptions of ISs make it difficult to replicate studies or use them in meta‐analyses and reviews (Bunger et al. 2017; Leeman et al. 2015). To enable the wider adoption of effective methods, there is an urgent need to meticulously document ISs and build a stronger foundation for specific strategies (Powell et al. 2015; Lewis et al. 2016).

To standardise practices, the Expert Recommendations for Implementing Change (ERIC) project has gathered expert consensus on terminology, definitions, and categories to guide implementation research (Powell et al. 2015). The ERIC project's panel of experts came to an agreement on a final list of 73 distinct IS, grouped into nine categories. These have been clustered as the following: (1) involving consumers, (2) using evaluation and interactive methods, (3) modifying infrastructure, (4) tailoring to specific contexts, (5) building stakeholder relationships, (6) employing financial strategies, (7) supporting healthcare providers/clinicians, (8) providing interactive support, and (9) training and educating stakeholders (Waltz et al. 2015). The project recommended that implementation science researchers use this comprehensive list of ISs, in combination with contemporary reporting guidelines, to improve the precision and clarity in documenting and reporting implementation strategies (Powell et al. 2015).

## 3 Methods

### 3.1 Study Setting

This study took place in the context of an enhanced primary care youth mental health programme. The Jigsaw model of therapeutic support is a brief, goal‐focused, transdiagnostic and solution‐oriented approach (Zeig and Gilligan 1990; de Shazer et al. 2007) designed for young people aged 12–25 years experiencing mild to moderate mental health difficulties. The model is grounded in principles of early intervention and enhanced primary care, emphasising timely, youth‐centred, and evidence‐informed support embedded within community settings (WEF 2020; McGorry et al. 2024).

At the time of writing, clinical assessment followed two structured stages. An Initial Screen identified presenting issues, risk and service suitability, followed by a more detailed HEADSS assessment exploring mental health, strengths, and support systems. Risk screening and goal setting were integrated throughout. Using this information, clinicians developed a collaborative formulation that reflects the young person's perspective and guides intervention planning. Interventions are brief, collaborative and focused on self‐identified goals. Support is delivered in person, via video, or by phone, depending on need and preference. Approaches include solution‐focused, cognitive‐behavioural and acceptance‐based methods.

Services are delivered by transdisciplinary teams, namely clinical, counselling, and educational psychologists, social workers, psychotherapists, mental health nurses and occupational therapists, operating within a shared governance structure that includes clinical supervision and case review. Core competencies include youth‐centred communication, collaborative goal‐setting, brief intervention, risk management and reflective practice.

Outcome measurement is integral to the model. Young people complete the CORE‐10 (ages 17+) or YP‐CORE (ages 12–16) and Goal‐Based Outcome Measures at key points in therapy. Data are recorded in the national electronic health record to monitor outcomes locally and nationally. In evaluation studies, young people engaging in Jigsaw demonstrated significant reductions in psychological distress following brief interventions, with the majority achieving reliable change on validated outcome measures (O'Reilly et al. 2016; Moore et al. 2025). Improvements were also observed in progress towards self‐identified goals, with nearly four‐fifths of participants reporting meaningful movement towards their therapy targets (O’Reilly et al. 2022).

Quality and safety are maintained through clinical supervision, weekly case‐review meetings, and routine clinical audits assessing the quality of documentation, goals, and risk management. Service‐level indicators such as wait times, attendance and outcomes are tracked through a national EHR, enabling continuous feedback and quality improvement. Youth participation is embedded at each service, with youth panels contributing to service design, evaluation, and continuous improvement to ensure that care remains developmentally and culturally relevant.

**FIGURE 1:**
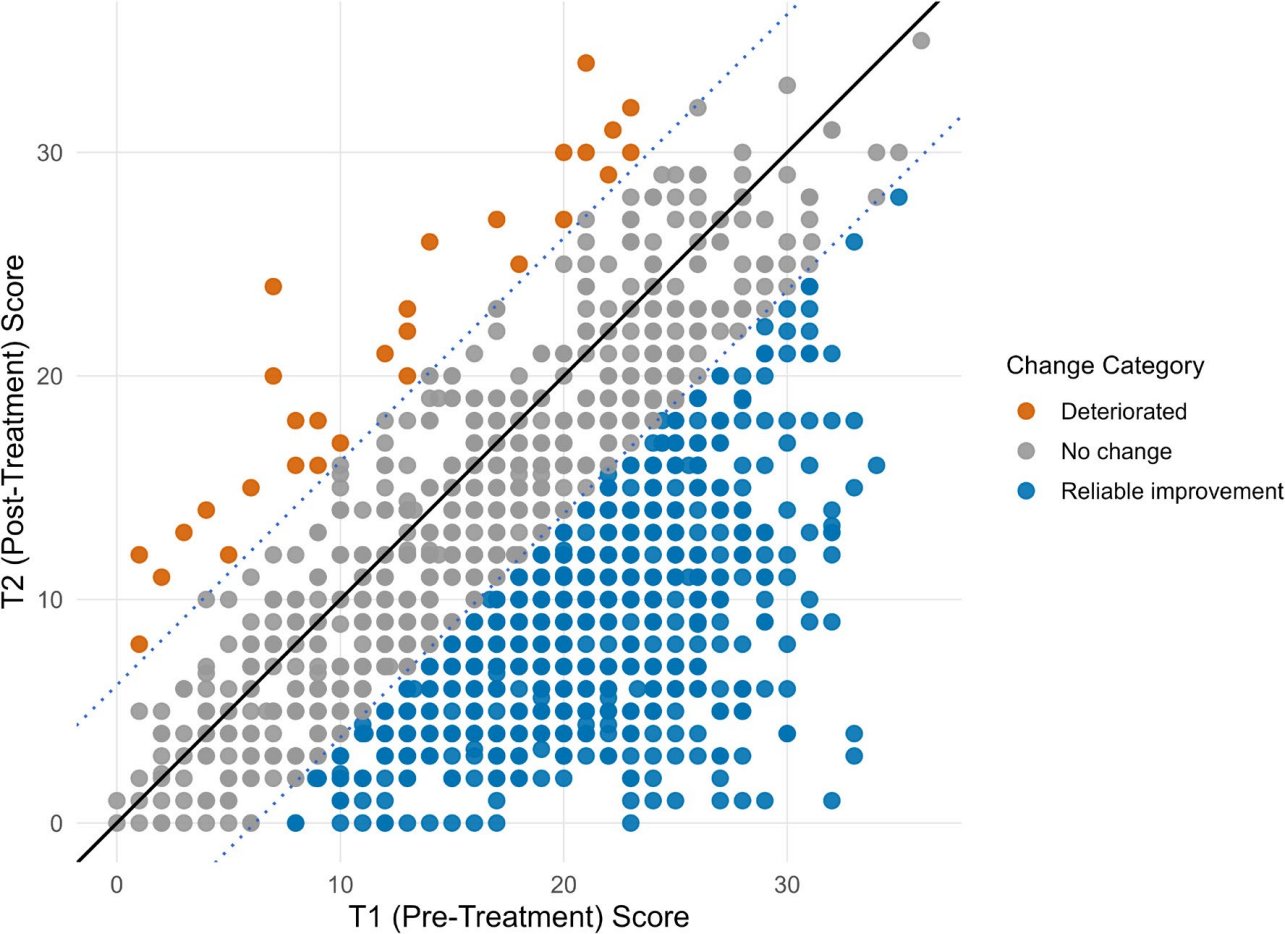
Plotting clinical outcomes for youth aged 12–16 in 2022.

Table 1 provides an overview of the key service metrics for 2022. Most young people attending Jigsaw report moderate to severe clinical levels of psychological distress pre‐intervention (O’Reilly et al. 2015). Clinical outcomes are measured using the widely deployed Clinical Outcome Routine Evaluation (10 and YP). The majority of young people report significant reductions in this distress following a brief intervention (O'Keeffe 2015; Moore et al. 2025). Figure 1 illustrates change across the therapeutic intervention for young people aged 12–16 (using the YP‐CORE). Over two‐thirds of young adults and 61% of young people achieved a reliable improvement in psychological distress after attending Jigsaw.

**TABLE 1:** Key metrics for the delivery of Jigsaw (2022).

	*n*	%	M (range)
Referrals			
Total referrals	8405	100.00	
Service offered	6688	79.57	
Clinical appointments offered	36 361		
Clinical appointments attended	27 961	76.91	
Referral source			
Parent	5150	62.21	
Self	1680	19.93	
GP	655	8.01	
Characteristics			
Female	5316	63.24	
Male	2897	34.47	
Unsure/questioning	193	2.30	
Age			16 (12–25)
Ethnicity			
White Irish	2582	83.29	
Black minority ethnic	222	9.07	
Presenting issues			
Anxiety	2888	68.00	
Low mood	1719	40.51	
Stress	1465	34.50	
Time waiting on care (weeks)—IQR			13 (9–18)
Session type			
In person	2754	75.74	
Video	7748	21.30	
Intervention type			
Brief intervention (1–8 sessions)	3866	49.61	
Brief contact (1–2 sessions)	2121	27.10	
Case consultation	1824	23.30	
Goal setting			
Reliable improvement in goals	2079	73.21	
Outcomes			
Young person core—reliable change	934	61.08	
Core 10—reliable change	538	66.41	

*Note:* Percentages are calculated from the number of cases with available data. Some categories allow more than one response per person, which is why totals do not sum to 100%.

### 3.2 Data Collection

Data collection took place in 2022. Three staff involved in designing and delivering ISs across the programme reviewed the ERIC refined compilation list to identify core strategies used in Jigsaw services in 2022. All three staff were involved in selecting implementation strategies for this and previous years. The lead author first reviewed a shared services log and the implementation blueprint to identify the implementation strategies used in Jigsaw during 2022. Each of the three project personnel then reviewed the ERIC refined compilation of 73 strategies and rated each one as either used (1) or not used (0) in Jigsaw. We use Fleiss' Kappa inter‐rater agreement to assess the level of agreement among the three raters' classifications of implementation strategies used during 2022. We found an inter‐rater agreement of 0.91, indicating excellent agreement among raters (Fleiss et al. 2003). Like other studies in this field (Moore et al. 2021), the group used a consensus‐building approach to agree on a final list of ISs used in Jigsaw from the ERIC. Although the clinical governance framework includes routine quality assurance and improvement activities such as supervision, audit, and performance monitoring, these differ in purpose from implementation strategies. Quality assurance mechanisms focus on service quality and local performance, whereas implementation strategies are deliberate, theory‐informed actions to support uptake, fidelity and sustainment (Bauer et al. 2020; Nilsen et al. 2022). In this study, quality assurance processes were examined as implementation strategies only when intentionally structured to achieve implementation outcomes (Proctor et al. 2013; Rudd et al. 2020; Bierbaum et al. 2025). Once the compilation of strategies had been identified and compiled, we set about documenting the operational domains of the reporting tool (Proctor et al. 2013). Using the Pragmatic Implementation Strategy Reporting Tool (Rudd et al. 2020; Perry et al. 2019), we described the operational elements (1) the actors; (2) the action(s); (3) the target(s) of the action; (4) temporality; (5) dose; (6) the implementation outcome(s) affected; (7) justification. In specifying these domains, we consulted a shared services calendar (which is a log of all events and activities), individual staff calendars and human resourcing records to specify dosage and actors. Like Bunger et al. (2017) in reporting dosage, we also collected data on the number of hours spent on different ISs per month. Where details on staff involvement, time or purpose were not clear from these materials, the lead author collected information directly from staff involved in implementation activities. Person‐hours were calculated by multiplying the duration of each activity by the number of staff involved, including both group meetings and individual work outside meetings as recorded in service logs. This study involved the secondary analysis of de‐identified administrative data routinely collected for service delivery purposes. As there was no direct participant contact, no new data collection and no foreseeable risk of harm, the study was deemed exempt from formal ethical review under institutional and national guidelines.

### 3.3 Data Analysis

All authors reviewed the resulting database of ISs before final analysis. Like Bunger et al. (2017), we used univariate techniques to describe the strategy types, temporality, dosage and actors. As one of our aims was to support future replication, we conducted a linear regression to predict dosage via person hours invested across implementation types and how well ISs types predicted time investment. In writing up the final report, we cross‐checked standards for reporting implementation studies (StaRI) (Pinnock et al. 2017).

## 4 Results

### 4.1 Types of Implementation Strategies

Table 2 summarises the 30 implementation strategies used in 2022. We identified no ISs in the domains of change infrastructure. We identified six individual ISs in the domains of both evaluative and iterative strategies and stakeholder engagement, representing 20% of all strategies. This was followed by the domain of training and education with five individual strategies. In terms of implementation outcomes, 16 of the ISs employed focus on ensuring programme fidelity and 15 are specifically aimed towards programme sustainment. In terms of the rationale for strategies, 50% of strategies (*n* = 15) had an empirical rationale.

**TABLE 2:** Compilation of implementation strategies (*n* = 30) employed in sustained Jigsaw services (2022).

	Hours	% of total hours	Staff (*n*)
Evaluative and iterative strategies			
Audit and provide feedback	1492	12.81	17
Communicate impact with stakeholders	20	0.17	7
Develop a formal implementation blueprint	20	0.17	6
Develop and organise tools for quality monitoring	13	0.11	4
Obtain and use service user feedback	360	3.09	28
Purposely re‐examine the implementation	36	0.31	5
Provide interactive assistance			
Centralised technical assistance	242	2.08	12
Facilitation	30	0.26	12
Provide supervision and line management	4020	34.52	18
Provide local technical assistance	1800	15.46	28
Adapt and tailor			
Promote adaptability	20	0.17	2
Use data experts	96	0.82	2
Use data warehousing	192	1.65	2
Stakeholder engagement			
Build an implementation coalition	384	3.30	12
Capture and share local knowledge	6	0.05	6
Develop an academic partnership	16	0.14	2
Implementation team meetings	120	1.03	40
Involve executive boards	16	0.14	2
Recruit, designate and train for leadership	480	4.12	3
Train and educate strategies			
Educational Meetings	40	0.34	1
Conduct ongoing training	96	0.82	6
Use train‐the‐trainer strategies	132	1.13	2
Create a learning collaborative	162	1.39	
Shadow experts	60	0.52	
Support clinicians			
Create new clinical teams	1272	10.92	5
Facilitate relay of clinical data to providers	4	0.03	4
Revise professional roles	120	1.03	5
Engage consumers			
Involve patients/consumers and family members	336	2.89	28
Use mass media	48	0.41	2
Financial			
Accessing new funding	12	0.10	3

### 4.2 Variation by Actors, Context and Outcomes

The context for most ISs was the national office (*n* = 24) insofar as the strategy was either designed by staff working in national support or oversight roles. However, some of the most time intensive strategies occurred in local services (e.g., supervision). Regional clinical and regional service managers were the most frequent actors in terms of IS (*n* = 16 strategies). Research and evaluation staff were the main actor in five ISs, as were local clinical and service managers. Clinician behaviour and practice was the target of most strategies, although some also targeted behaviour of managers and service users. In terms of implementation outcomes, over half were aimed at sustainment (*n* = 15, 51.71%), followed by fidelity (26.88%, *n* = 8).

Table 3 details dosage by implementation strategy categories. We estimated the person‐hours (representing one hour of work by one person) invested in implementation in total, and over time. When the number of individuals participating in each activity is considered, implementation activities accounted for 11 645 person‐hours (about 199 8‐h workdays) over the observation period. We examined the dosage of implementation efforts based on the number of staff involved in implementation and the total amount of time spent on each strategy. Four of the strategies took place annually, such as some evaluative strategies (such as clinical audits), and five on a quarterly basis. Five of the identified strategies took place weekly. ISs like facilitation took place on an ad hoc basis and training was scheduled across the year (and specifically not scheduled for staff holiday periods over the summer). Interactive assistance was the most labour intensive, with over half of the time recorded spent on this strategy (6092 h). Jigsaw provides staff with both clinical supervision and line management and this accounts for a sizable proportion of this domain. Nearly 20% of our time spent on implementation activities was allocated towards iterative and evaluative strategies, with routine case note audits and collection and monitoring of user feedback taking the most time.

**TABLE 3:** Strategy dosage by category.

Implementation strategy	*n*	Actors involved	Person—hours	
		Range (M)	Total hours	%
Iterative and evaluative strategies	6	3–28 (11.16)	1941	17
Train and educate	5	18–28 (10.03)	490	4
Engagement consumers	2	2–28 (15.21)	384	3
Tailor and adapt	3	2 (2.01)	308	3
Stakeholder engagement	6	4–40 (10.83)	1022	9
Provide interactive assistance	4	12–28 (17.50)	6092	52
Change infrastructure	0	n/a	n/a	0
Support clinicians	3	4–6 (4.61)	1396	12
Financial	1	3	12	0

To enhance interpretability and support replication, Table 4 summarises the highest‐use implementation strategies identified in 2022. In addition to quantifying staff time, it describes how these strategies were enacted in practice, outlining the actors involved, their typical formats, and how each activity functioned to support fidelity, sustainment, or improvement. Together, these examples illustrate how supervision, technical assistance, and evaluative feedback were operationalised as core mechanisms sustaining model delivery across sites.

**TABLE 4:** Highest use implementation strategies.

Implementation strategy	Staff involved	Total hours	Description of delivery in practice
Provide supervision	Regional clinical managers and clinical managers	4024	Clinicians received both clinical and line management supervision through structured frameworks. Formats included individual, group, and discipline‐specific supervision. Supervision policy recommends Proctor's three‐function model (Proctor 1987); Gibb's Reflective Cycle (Gibbs 1988) and Hawkins and Shohet's Seven‐Eyed Model (Hawkins and Shohet 2012). A naturalistic, observational study of supervision across the programme was conducted in 2021 (Oostermeijer et al. 2021).
Provide local technical assistance	Clinical and services managers	1800	Managers provide on‐demand support, advice and consultation to staff in local sites. Weekly meetings and real‐time support addressed implementation challenges, ensuring consistency and fidelity across sites.
Audit and provide feedback	Regional clinical managers, evaluation team	1492	Annual national audits of clinical case notes assessing assessment, formulation, risk management, goal setting, and documentation quality. Feedback was shared in supervision and team meetings to promote fidelity and continuous improvement. Monthly spot check audit conducted by clinical managers for each clinician.
Centralised technical assistance	National staff; regional clinical & service managers	720	Site‐level implementation support tailored to local needs through scheduled and ad hoc facilitation, problem‐solving, and guidance on policy and practice alignment.
Create new clinical teams	Regional and senior managers; HR staff	1272	Coordinated recruitment and onboarding for new transdisciplinary teams. Processes emphasised balanced professional representation, shared governance, and orientation to the service model to ensure consistency and readiness for implementation.
Build an implementation coalition	Service director, clinical director, regional managers	384	Fortnightly leadership and management forums used to coordinate implementation priorities, align strategy, and share learning across regions and services.
Obtain and use service user feedback	Service administrators, clinicians, managers	360	Service administrators and clinicians collected routine feedback using the *Youth Service Satisfaction Survey* (Doyle et al. 2024) and parent feedback measure. Data on service experience and acceptability were reviewed monthly by local managers and discussed in supervision and team meetings to inform ongoing quality improvement. Feedback was also analysis and reported nationally.

**FIGURE 2:**
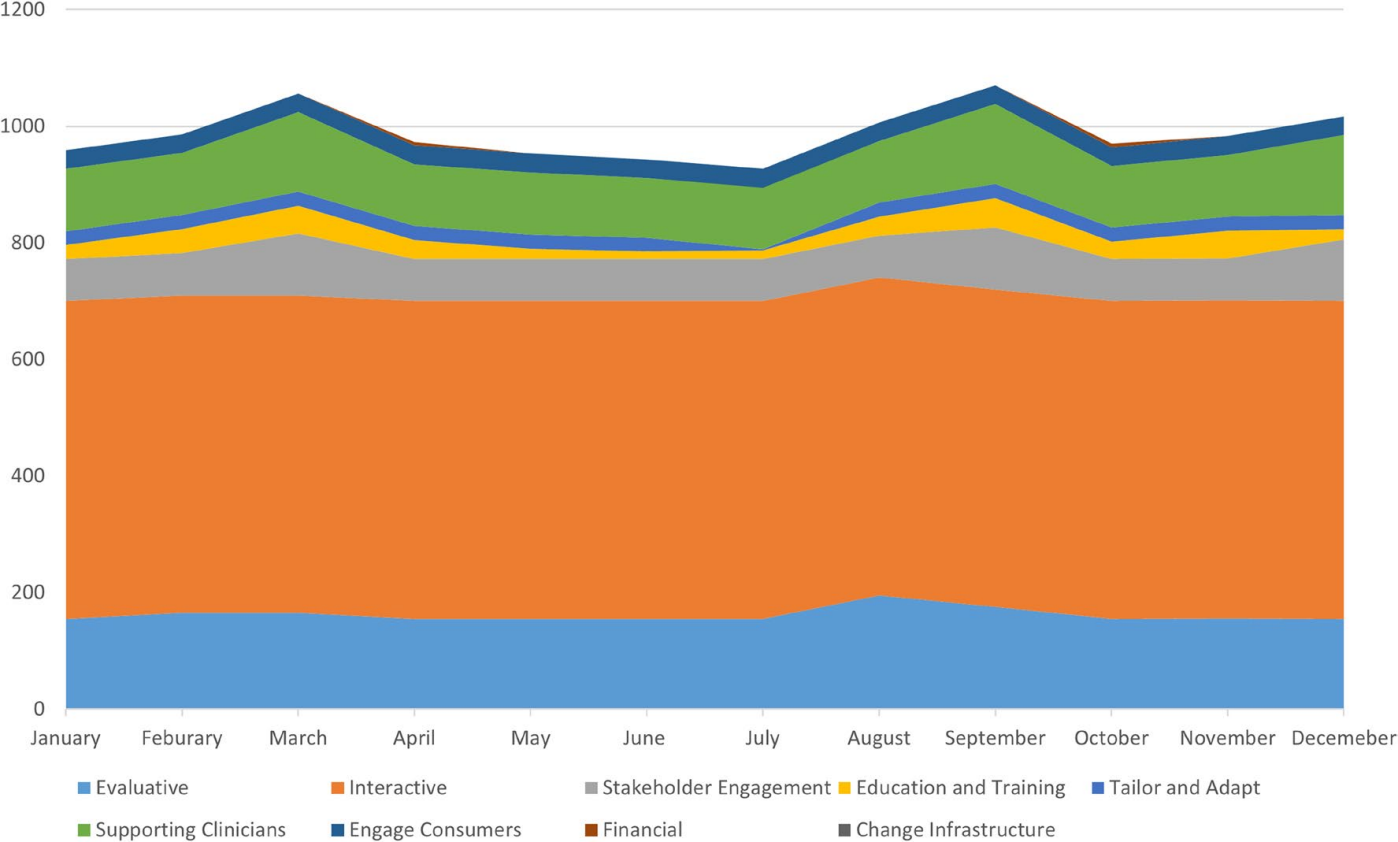
Number of hours invested across implementation strategy domains (2022).

Figure 2 illustrates the timing of these strategies over the course of the calendar year. Strategies like interactive assistance, local technical assistance and evaluative and iterative strategies were consistent in terms of inputs across the year. Others like stakeholder engagement, education and training and consumer engagement spiked in March and September. This mirrors trends in service delivery with decreases in demand during summer months and reduced staff availability during summer months.

Table 5 details the linear regression predicting investment in hours. The model shows that only interactive assistance was a significant predictor of increased time investment. For every one‐standard‐deviation increase in interactive assistance, the model shows an increase in time investment by 0.55 standard deviations. The other variables did not have statistically significant effects on time. A power analysis result of 0.64 for the linear regression indicates moderate statistical power.

**TABLE 5:** Regression results—association between implementation strategy type and dosage.

	*B*	SE	*β*	*t*	*p*
Interactive assistance	1320.00	490.81	0.55	2.69	0.01
Adapt and tailor	−220.83	537.65	−0.08	−0.41	0.69
Stakeholder engagement	−153.17	438.99	−0.08	−0.35	0.73
Train and educate	−208.00	490.81	−0.09	−0.42	0.68
Supporting clinicians	141.83	537.65	0.05	0.26	0.79
Consumer engagement	−131.50	620.83	−0.04	−0.21	0.83
Finance	−311.50	821.28	−0.07	−0.38	0.71

## 5 Discussion

This paper is one of the first attempts to employ contemporary reporting techniques to describe the ISs used to facilitate the delivery of an primary care youth mental health programme. Criticisms of the field of implementation science include that most studies focus on a limited number of ISs (Waltz et al. 2021). In our comprehensive retrospective review involving on‐the‐ground implementers, we identified 30 ISs used, illustrating the complexity and multi‐faceted nature of the ISs used in delivering a programme of this scale. These findings echo similar studies exploring ISs across several primary care mental health settings employing an average of 26 IS in sustainment (Waltz et al. 2021).

Our findings show that over 11 000 h plus of implementation activity were required to sustain a network of over 190 clinicians providing nearly 30 000 appointments over one calendar year. A study examining the implementation of person centred care in Sweden across 11 sites reported 11 076 person‐hours across all units (Fridberg et al. 2022). Tracking ISs in this way helps us understand how much time is invested in implementation or what it takes (outside of direct delivery with clients) to support programmes. This approach can help in resource planning and costing, but it does not necessarily evidence which individual strategies are most effective in adoption or sustaining interventions. Studies which assess the effectiveness of different strategies, individually or in combination, are needed to help inform the selection of strategies for future implementation.

Interactive assistance was by some distance the most time‐consuming strategy area in Jigsaw. Interactive assistance also predicted the highest time investment, reflecting the supervision and support needed to sustain fidelity across a large workforce. The regression was intended to examine patterns of resource use rather than effectiveness. This concentration of effort underscores the importance of relational strategies in sustainment. Although this study cannot speak to cause and effect, the level of time and attention devoted to supervision and local technical support suggests that these high‐demand strategies are at the heart of how the service is sustained. Their prominence also hints that these more relational, hands‐on forms of support may matter most during ongoing programme maintenance, though further research would be needed to understand their specific contribution to outcomes.

Supervision functioned as a high‐intensity implementation strategy, delivered through structured one‐to‐one sessions that developed clinician skill, reinforced adherence to the model, and provided a protected space for reflective and adaptive problem‐solving. In line with Proctor's three‐function framework (Proctor 1987) and informed by established reflective models Gibbs (1988) and Hawkins and Shohet (2012), this supervisory structure acted as an ongoing mechanism for maintaining fidelity, stabilising clinical decision‐making, and supporting consistent practice quality across a large, distributed workforce. In fact, the levels of support and good quality clinical supervision are commonly reported as a facilitator by Jigsaw staff. This finding is at odds with recent expert assessments of the importance and feasibility of the ERIC implementation compilation, which identified evaluation as the most important strategy (Waltz et al. 2015). Taken together, our findings suggest that interactive assistance plays a central role during the sustainment phase of IYS programmes. The main informants were staff involved in the design and delivery of ISs, and this is a limitation, as is the retrospective nature of the data collection. We collated information about implementation activities through review of a national log/database, calendars and via retrospective information from some staff. By failing to include front‐line workers as informants in the study, we may have missed ad hoc activities (such as work between meetings), and we were not able to report on any local variations in implementation strategies. To ensure more reliable data, we recommend collecting data on implementation activities from the earliest stages and on an ongoing basis in real time. At the outset, we decided to report only on ISs in use in 2022; we hope this leads to a more accurate reflection of implementation during this phase. However, it also resulted in some strategies that were critical in the early scaling up of this network not being reported.

## 6 Conclusion

Using implementation report guidelines, this study summarised the ISs used during 1 year across a programme of youth mental health. Our results show multiple and complex strategies, with interactive assistance and evaluation being especially important. IS reporting tools can help organisations understand and plan for future implementation.

## Funding

This work was supported by the Health Service Executive (Ireland).

## Conflicts of Interest

All authors were employed by Jigsaw at the time of writing. The Health Service Executive in Ireland funds Jigsaw service delivery and research and evaluation programme. The authors declare no conflicts of interest.

## Data Availability

The data that support the findings of this study are available from the corresponding author upon reasonable request.
